# Oocyte Penetration Speed Optimization Based on Intracellular Strain

**DOI:** 10.3390/mi13020309

**Published:** 2022-02-17

**Authors:** Yaowei Liu, Maosheng Cui, Yidi Zhang, Xiangfei Zhao, Mingzhu Sun, Xin Zhao

**Affiliations:** 1 Institute of Robotics and Automatic Information System, The Tianjin Key Laboratory of Intelligent Robotics, Nankai University, Tianjin 300071, China; liuyaowei@mail.nankai.edu.cn (Y.L.); yidi_zhang@mail.nankai.edu.cn (Y.Z.); 1120170124@mail.nankai.edu.cn (X.Z.); sunmz@nankai.edu.cn (M.S.); 2 Institute of Intelligence Technology and Robotic Systems, Shenzhen Research Institute of Nankai University, Shenzhen 518083, China; tjsnykxyxmsyyjs@tj.gov.cn; 3 Institute of Animal Sciences, Tianjin 300112, China

**Keywords:** robotic cell manipulation, cell penetration, intracellular strain, cell development potential

## Abstract

Oocyte penetration is an essential step for many biological technologies, such as animal cloning, embryo microinjection, and intracytoplasmic sperm injection (ICSI). Although the success rate of robotic cell penetration is very high now, the development potential of oocytes after penetration has not been significantly improved compared with manual operation. In this paper, we optimized the oocyte penetration speed based on the intracellular strain. We firstly analyzed the intracellular strain at different penetration speeds and performed the penetration experiments on porcine oocytes. Secondly, we studied the cell development potential after penetration at different penetration speeds. The statistical results showed that the percentage of large intracellular strain decreased by 80% and the maximum and average intracellular strain decreased by 25–38% at the penetration speed of 50 μm/s compared to at 10 μm/s. Experiment results showed that the cleavage rates of the oocytes after penetration increased from 65.56% to 86.36%, as the penetration speed increased from 10 to 50 μm/s. Finally, we verified the gene expression of oocytes after penetration at different speeds. The experimental results showed that the totipotency and antiapoptotic genes of oocytes were significantly higher after penetration at the speed of 50 μm/s, which verified the effectiveness of the optimization method at the gene level.

## 1. Introduction

The oocyte penetration is an essential step in many biological technologies, such as somatic cell nuclear transfer (SCNT) [1,2,3,4,5,6,7], embryo microinjection [8,9,10], and intracytoplasmic sperm injection (ICSI) [11,12,13,14]. Improving the oocyte development potential after penetration operation can directly improve the cell development potential of these complex biological cell experiments with penetration operation. Although the success rate of robotic cell penetration is very high now, the development potential of oocytes and embryos after being penetrated has not been significantly improved compared with manual operation. For example, the success rate of injection of robotic ICSI by Lu et al. has reached 90%, but the subsequent cell development potential is not improved compared with manual results [12]. Mattos automatically injected the blastocyst, which resulted in a 20% yield of chimeras that was slightly lower than the manual blastocyst microinjections (22.2%) [15]. Therefore, it is undoubtedly necessary to improve the development potential after penetration.

During the process of oocyte penetration, the oocytes suffer from large deformation and lead to high intracellular strain, which may cause damage to the oocytes and may eventually lead to the reduction of oocyte development potential. Many studies have shown that large intracellular strain can cause potential damage to cells and reduce the development potential of cells. For example, Scott et al. found that short-term, high-strain mechanical loading may lead to apoptosis [16]; S. J. Gladman also mentioned that the mechanical injury (20% tensile strain) led to significant neuronal cell death [17]. In our previous studies, we have also found that there is a strong negative correlation between intracellular strain and cell development potential [18]. Therefore, reducing the intracellular strain in the process of oocyte penetration may increase the oocyte development potential after penetration.

Meanwhile, some researchers found that the penetration speed could affect the cell development potential. For example, Liu et al. used the number of ruptured bonds to describe the damage of the cell during microinjection and found that the larger velocity caused fewer number of ruptured bonds, which might result in better development potential [19]. As the intracellular strain and the penetration speed are all negatively correlated with the cell development potential, it is feasible to optimize the penetration speed based on the intracellular strain.

In this paper, we optimized the oocyte penetration speed based on the intracellular strain. Firstly, we analyzed the intracellular strain under the condition of different penetration speed and then performed the penetration experiments on oocytes at different penetration speed. We used the optical flow method to detect the intracellular strain of each oocyte during the penetration process and obtained the penetration speed that can minimize the intracellular strain and ensure the accuracy: 50 μm/s. Secondly, we studied the cell development potential after penetration at different penetration speeds. We penetrated the oocytes with the speed of 10, 20, and 50 μm/s. The results showed that the optimized penetration speed improved the cleavage rate from 65% to 86%. Finally, we verified the gene expression of oocytes after penetration at different speeds. The experimental results showed that the totipotency and antiapoptotic genes of oocytes were significantly higher after penetration at the speed of 50 μm/s, which verified the effectiveness of the optimization speed at the gene level.

## 2. Materials and Methods

### 2.1. Oocyte Preparations

We collected porcine ovaries from a local slaughterhouse and aspirated ovary follicles with diameter a between 3–6 mm using a sterile 10 mL syringe with an 18-gauge needle to acquire the cumulus–oocyte complexes (COCs). After washing three times in Tyrode’s lactate (TL)-Hepes-PVA (polyvinyl alcohol, 0.1%), 30 COCs were transferred into 100 μL drops of maturation medium (TCM-199 supplemented with 15% FBS, 10 ng/mL EGF, 10% porcine follicular fluid, 10 IU/mL of eCG, 5 IU/mL of hCG, and 0.8 mM L-glutamine and 0.05 mg/mL gentamicin) for 42 h at 38.5 °C, 5% CO_2_, and saturated humidity. After maturation culture, we picked oocytes with clear perivitelline spaces and intact cell membranes and extruded the first polar body (PB1) for use.

### 2.2. Activation of Oocytes

As the cleavage rate could represent the development potential of the oocytes after cell operation [20,21], we need to calculate the cleavage rate of the oocyte at 48 h after oocyte penetration experiment and parthenogenetic activation. The oocytes after penetration experiments were activated simultaneously by the application of one DC electric pulses of 1.25 kv/cm for 50 μs in an activation–fusion medium (0.28 M mannitol, 0.5 mM HEPES, 0.01% BSA, 1 mM CaCl_2_, and 0.1 mM MgCl_2_). The activation/fusion embryos were selected and transferred to fresh PZM-3 for further culture under 38.5 °C, 5% CO_2_, and saturated humidity.

### 2.3. Intracellular Strain Calculation

The intracellular strain field of the oocyte was obtained using Equation (1):

(1) $$\varepsilon =\underset{L\to 0}{\lim}\frac{\Delta L}{L}$$

where *L* is the initial distance between two adjacent points and Δ*L* is the distance variation after deformation. Since the deformation and strain of the oocytes occurred mainly in the horizontal direction, we ignored the vertical deformation and calculated the strain field by Equation (2):

(2) $$\varepsilon =\frac{\left|x\left(i+1\right)-x\left(i\right)\right|-L}{L}$$

where *L* is the horizontal distance between two adjacent points in one frame, while *x*(*i*) and *x*(*i* + 1) are the horizontal positions of the adjacent points in the next frame. The intracellular structure is stretched when strain is greater than zero, and the intracellular structure is compressed when strain is less than zero.

### 2.4. Real-Time PCR

Primers of Oct4, Rex01, and Bcl-2 genes for Real-Time PCR are shown in Table 1. The quantification of all gene transcripts was carried out in three replicates by quantitative real-time reverse transcriptase PCR on a GeneAmp 5700 using SYBRGreen PCR Master mix (Applied Biosystems, Tiangen, China). The reaction mixture of the total 50 μL volume consisted of 25 μL 2× master mix (containing SYBR Green 1 Dye, AmpliTaq DNA Polymerase, dNTPs with dUTP and optimized buffer components), 1 to 1.5 μL (0.2 to 0.3 μm) of each primer, 2 to 4 μL of cDNA template, and the remainder was Diethylpyrocarbonate (DEPC)-treated water. The comparative threshold cycle (CT) method was used for the quantification of expression levels.

### 2.5. Statistical Analysis

Rates of cleavage were firstly subjected to arcsine transformation and then analyzed by one-way ANOVA. Differences were determined by either one-way ANOVA followed by Fisher’s least-significant difference multiple comparison test for experiments with multiple groups, or a Student *t*-test for experiments with two treatment groups. For qPCR data, expression levels between treatments were determined using the comparative threshold cycle (CT) method for each gene. Data were reported as means ± SEM. *p* < 0.05 values were considered significant.

## 3. Results

### 3.1. System Setup

**Figure 1:**
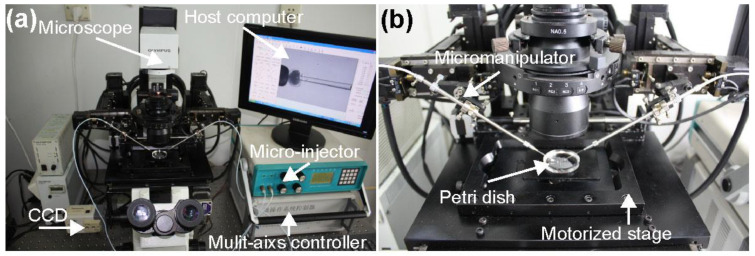
The NK-MR601 micro-manipulation system. (**a**) System setup; (**b**) micro operation workspace.

The oocyte penetration experiments were performed on the NK-MR601 micro-manipulation system [22,23,24], as shown in Figure 1. The system consists of an inverted microscope (CK-40, Olympus, Tokyo, Japan), a CCD camera (W-V-460, Panasonic, Osaka, Japan, frame rate: 20 frames/s), a motorized X–Y stage (travel range: 100 mm, repeatability: 1 μm/s, maximum speed: 2 mm/s), and two X–Y–Z micro-manipulators (travel range: 50 mm, repeatability: 1 μm/s, maximum speed: 1 mm/s); a micro-injector provides the negative pressure to hold the oocyte (−3~0 kPa); a motion control box controls the motorized stage, motorized micro-manipulators, and micro-injector through the host computer.

The holding micropipette (used to hold the oocyte) was made from a borosilicate glass tube with an outer diameter of 1 mm and an inner diameter of 0.6 mm. It was firstly pulled by the puller (MODEL P-97, Sutter Instrument, Novato, CA, USA) and then fractured by the microforge (MF-900, NARISHIGE, Tokyo, Japan). The holding micropipette was melted with an alcohol lamp by Yaowei Liu to smooth the tip opening. The diameter of the holding micropipette was about 50–80 μm. The injection micropipette was purchased from CooperSurgical (TPC, LBC-OD20BA90, Trumbull, CT, USA) with an outer diameter of 20 μm and a slope angle of 45 degrees.

### 3.2. Intracellular Strain under Different Penetration Speeds

The porcine oocytes were used in this paper. The penetration speed is usually set below 100 μm/s in oocyte penetration, in order to ensure the positioning accuracy of the micropipette in the oocyte. In oocyte penetration experiments, we used 60 oocytes in six groups and penetrated the oocytes by the injection micropipette at the speeds of 10, 20, 30, 40, 50, and 100 μm/s, respectively, after rotating the oocytes to the desired orientation (e.g., the polar body at 4 o’clock). The oocytes were collected in the same batch and cultured in the same condition.

**Figure 2:**
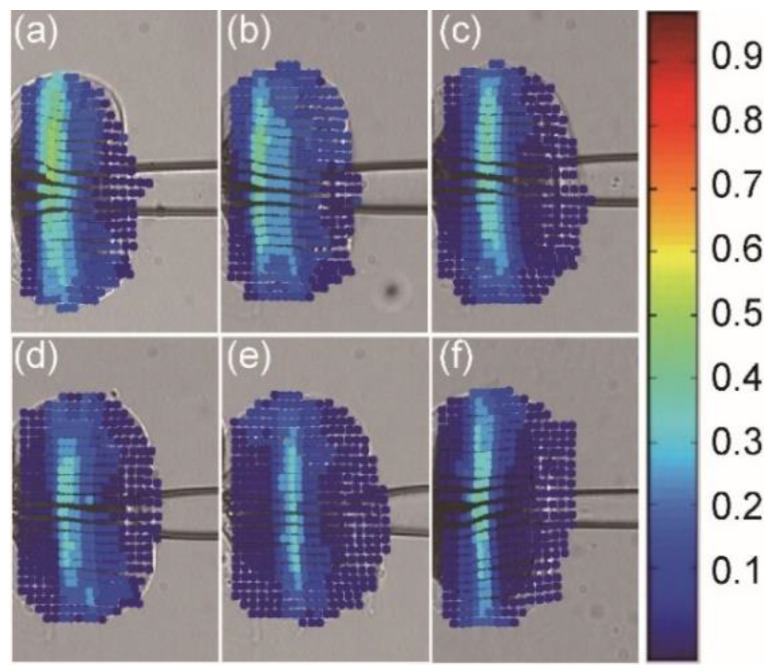
Intracellular strain field with the maximum deformation in the penetration process. From (**a**) to (**f**): penetration speeds of 10, 20, 30, 40, 50, and 100 μm/s. Red and blue represent the high and low values.

The strain distribution with the maximum deformation in each penetration process was illustrated in Figure 2. Videos S1–S6 show the penetration processes and corresponding strain distributions at the speeds of 10, 20, 30, 40, 50, and 100 μm/s, respectively. The results showed that the large intracellular strain value decreased, and its range shrank as the penetration speed increased.

**Figure 3:**
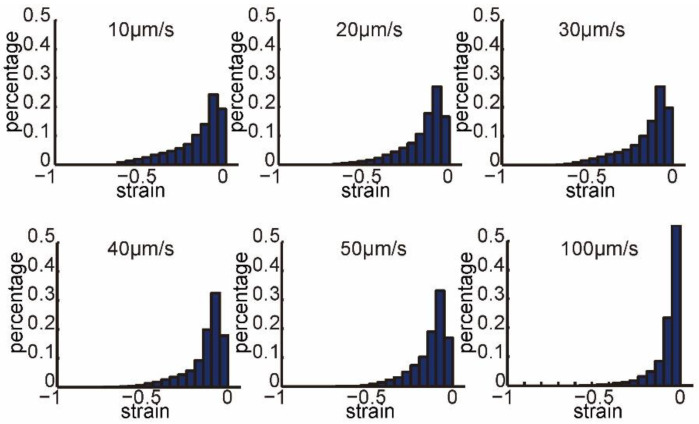
Normalized histograms of the strain field on all frames of 10 penetration processes at each penetration speed (penetration speed: 10, 20, 30, 40, 50, and 100 μm/s).

**Figure 4:**
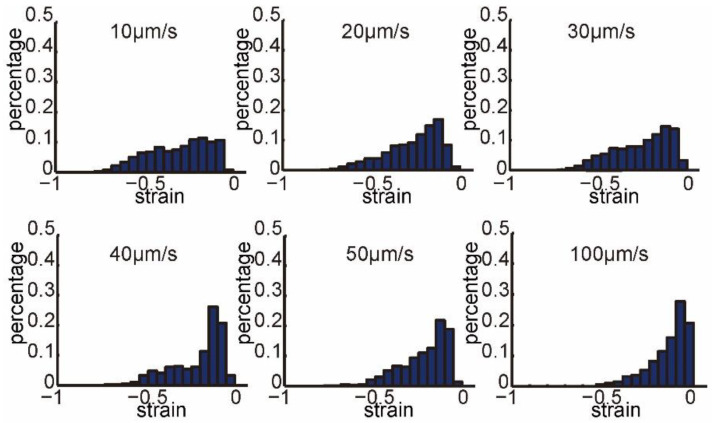
Normalized histograms of the strain field on the last frame before oocyte penetrated at each penetration speed (penetration speed: 10, 20, 30, 40, 50, and 100 μm/s).

Figure 3 and Figure 4 show the normalized histograms of the strain field at different penetration speeds. In each figure of Figure 3, we collected the strain values on all frames of 10 penetration processes at each penetration speed. Figure 3 shows that strain distributions at different penetration speeds had similar profile. The maximum values and percentages of large values decreased, when increasing penetration speed. There were 4.63%, 2.89%, 2.78%, 1.44%, 0.82%, and 0.04% of large strain values distributed in [−1.0, −0.5], when penetration speeds were 10, 20, 30, 40, 50, and 100 μm/s, respectively.

In each figure of Figure 4, we collected the strain values on the last frame before oocyte penetrated at each penetration speed. Figure 4 shows that strain distributions at different penetration speeds had different profiles, as both the maximum values and percentages of large values decreased greatly when increasing penetration speed. There were 18.11%, 10.87%, 10.44%, 5.71%, 3.52%, and 0.33% large strain values distributed in [−1.0, −0.5], when penetration speeds were 10, 20, 30, 40, 50, and 100 μm/s, respectively.

The statistical results showed that the percentages of the large intracellular strains at the penetration speeds of 50 and 100 μm/s were all below 1% in the penetration process and below 5% on the last frame, respectively, which indicated that the intracellular strain could be effectively reduced at the penetration speeds of 50 and 100 μm/s.

**Figure 5:**
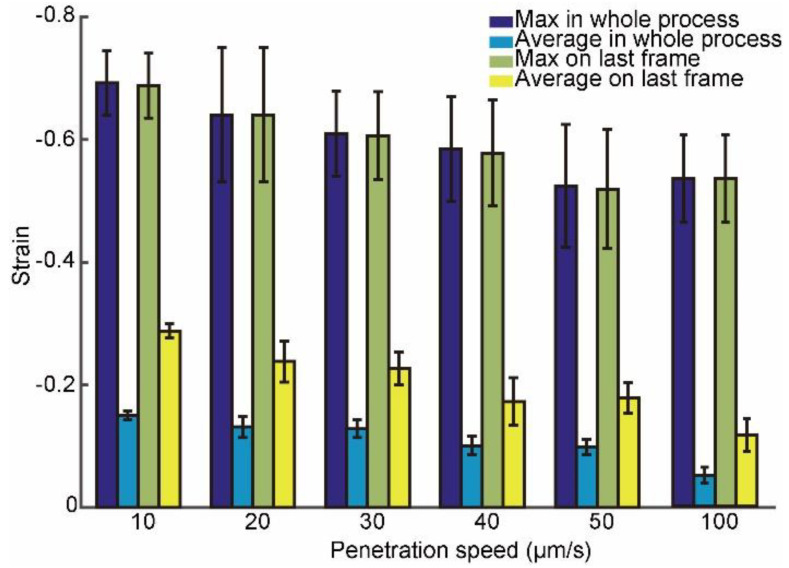
Maximum and average intracellular strains at different penetration speeds in the whole penetration process and on the last frame before oocyte penetrated.

Furthermore, we calculated the maximum and average of strain values at different penetration speeds, as shown in Figure 5. Calculation details were described in Appendix A. When the penetration speed was 10, 20, 30, 40, 50, and 100 μm/s, the maximum values in the whole penetration process were −0.69 ± 0.05, −0.64 ± 0.11, −0.61 ± 0.07, −0.58 ± 0.09, −0.52 ± 0.10, and −0.53 ± 0.10 (*n* = 10); the average strain values were −0.15 ± 0.01, −0.13 ± 0.02, −0.13 ± 0.02, −0.10 ± 0.01, −0.10 ± 0.01, and −0.05 ± 0.01 (*n* = 10); the maximum strain values on the last frame before oocyte penetrated were −0.69 ± 0.05, −0.64 ± 0.11, −0.61 ± 0.07, −0.58 ± 0.09, −0.52 ± 0.10, and −0.53 ± 0.10 (*n* = 10); and the average strain values were −0.29 ± 0.01, −0.24 ± 0.03, −0.23 ± 0.03, −0.17 ± 0.04, −0.18 ± 0.03, and −0.11 ± 0.03 (*n* = 10). The statistical results indicated that the maximum and average strains at the penetration speeds of 50 and 100 μm/s were similar and decreased greatly compared to those at 10 μm/s, which reduced potential intracellular damage greatly, since the large strains caused potential intracellular damage. Therefore, the penetration speed was set to 50 μm/s to ensure the positioning accuracy and reduce the cell damage at the same time.

### 3.3. Cell Development Potential at Different Penetration Speeds

**Figure 6:**
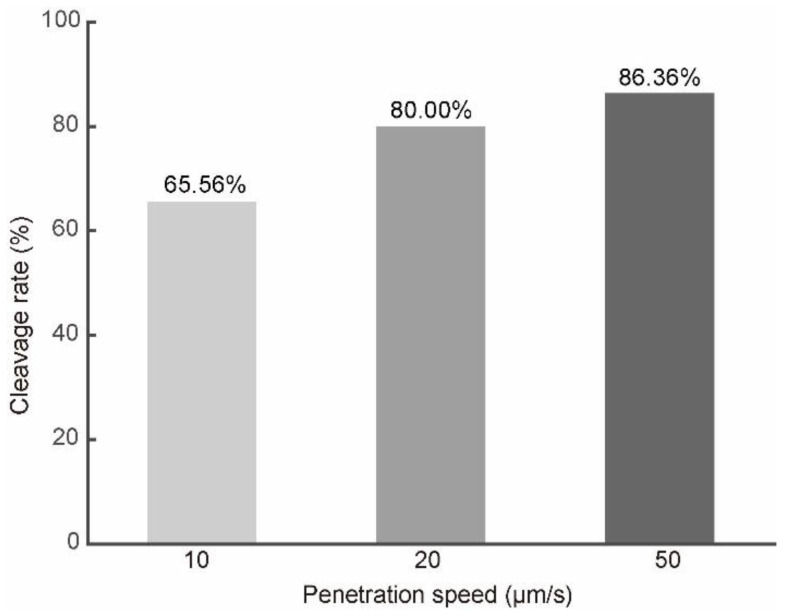
The cleavage rates of the embryos at different penetration speeds.

In order to verify that the development potential of porcine oocytes is actually increased after being penetrated at a speed of 50 μm/s, a total number of 268 oocytes in three groups were penetrated at the speeds of 10, 20, and 50 μm/s. All the oocytes were rotated to the same orientation before penetration (polar body at 4 o’clock in this paper). The oocytes were collected in the same batch and cultured in the same condition. Then, the oocytes were activated by applying one DC electric pulse of 1.2 kv/cm for 80 μs. The cleavage rate was used to evaluate the development potential of the porcine oocytes after penetration [20,21]. After two days culturing, 59 out of 90, 72 out of 90, and 76 out of 88 oocytes cleaved. The cleavage rates were 65.56%, 80.00%, and 86.36%, respectively, as shown in Figure 6. The statistical results indicated that the cleavage rate increased as the penetration speed increased. One reasonable explanation is that, as the penetration speed increases, the intracellular strain decreases, so the damage to the oocytes is reduced and the development potential increases.

**Figure 7:**
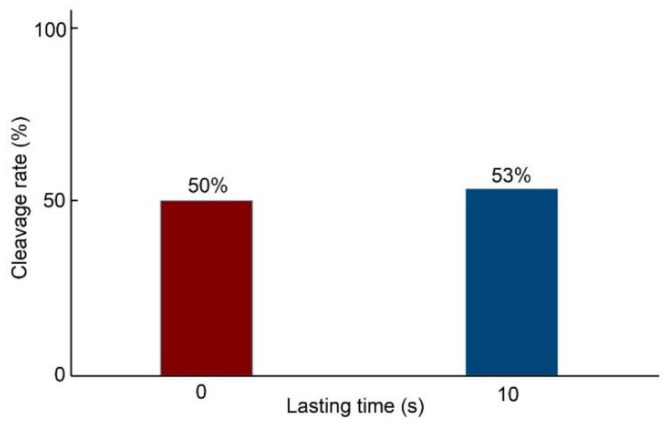
Cleavage rates of embryos in different lasting time groups.

Additional experiments were designed to exclude the influence of the force lasting time. In this experiment, we poked the oocytes at the speed of 50 μm/s under the same condition in the two groups. The oocytes were collected in the same batch and cultured in the same condition. The micropipette poked and left the oocytes immediately in Group One, while the micropipette kept poking for 10 s before leaving in Group Two. A total number of 60 oocytes was used in this experiment, and each group had 30 oocytes. The cleavage rates of embryos in group one and group two were 50% (15/30) and 53.3% (16/30), respectively, which indicated that the lasting time did not affect the development potential of the oocytes. Figure 7 gives the cleavage rates of embryos in different lasting time groups.

### 3.4. Gene Expressions under Different Penetration Speeds

A total of 960 oocytes in four groups were used to assay gene expressions of the embryos. The oocytes were collected in the same batch and cultured in the same condition. The detected genes were developmental totipotency genes (Rex01 and Otc4) and an antiapoptotic gene (Bcl-2). In this experiment, the 240 oocytes in the control group were treated as followed: collected, activated, and gene expressions assayed. The oocytes in other three groups were treated as followed: collected, penetrated at the speeds of 10, or 20, or 50 μm/s, and activated, and then gene expressions were assayed.

**Figure 8:**
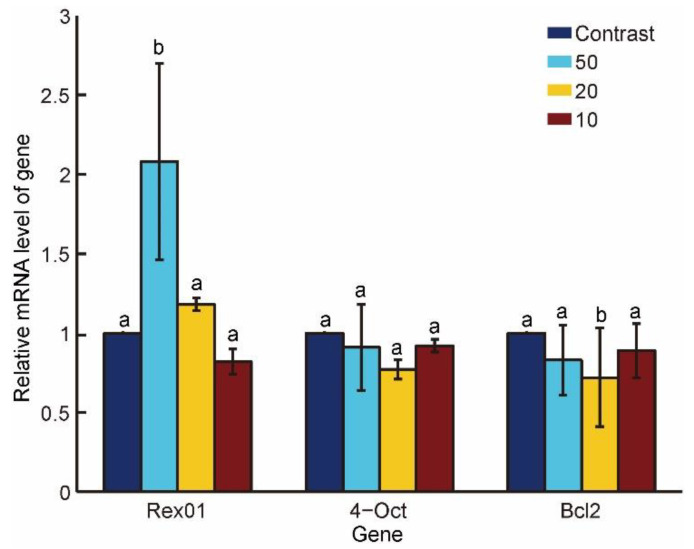
Gene expressions of the embryos at 17 h after oocytes been fused/activated. The Rex01 gene expression at the penetration speed of 50 μm/s was significantly higher than those of control, 20 μm/s and 10 μm/s (*p* < 0.05). The a and b in the figure mean the data in b group is significantly different from that of a.

Figure 8 shows the Rex01, Oct-4, and Bcl-2 gene expressions of the embryos at 17 h after oocytes being fused/activated, which revealed that the Rex01 gene expression at the penetration speed of 50 μm/s (2.08 ± 0.62) was significantly higher than those of the control (1.00 ± 0), 20 μm/s (1.18 ± 0.04), and 10 μm/s (0.82 ± 0.08), (*p* < 0.05).

**Figure 9:**
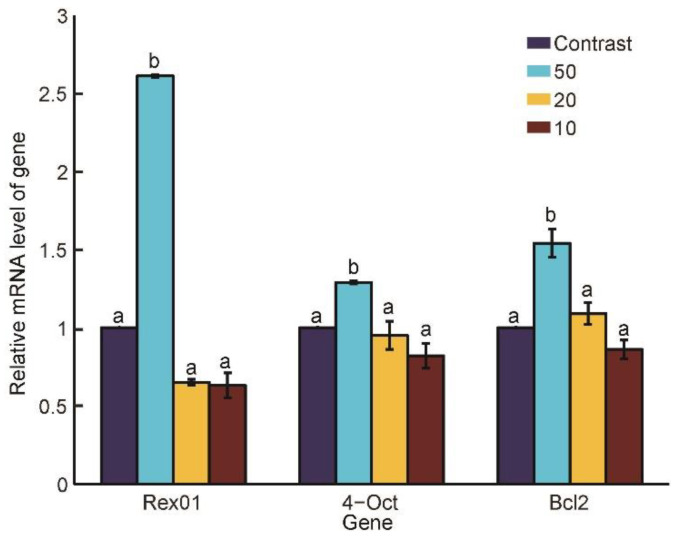
Gene expressions of the embryos at 72 h after oocytes been fused/activated. The Rex01, Oct-4, and Bcl-2 gene expressions at the penetration speed of 50 μm/s were all significantly higher than those of the control, 20 μm/s, and 10 μm/s (*p* < 0.05). The a and b in the figure mean the data in b group is significantly different from that of a.

Figure 9 shows the Rex01, Oct-4, and Bcl-2 gene expressions of the embryos at 72 h after oocytes being fused/activated in the four groups. The statistical results revealed that the Rex01 gene expression at the penetration speed of 50 μm/s (2.61 ± 0.01) was significantly higher than those of the control (1.00 ± 0), 20 μm/s (0.65 ± 0.02), and 10 μm/s (0.63 ± 0.08), (*p* < 0.05); the Oct-4 gene expression at the speed of 50 μm/s (2.61 ± 0.01) was also significantly higher than those of the other groups (1.00 ± 0 vs. 0.95 ± 0.09 vs. 0.82 ± 0.08), (*p* < 0.05); the Bcl-2 gene expression at the speed of 50 μm/s (1.54 ± 0.09) was significantly higher than those of the other groups (1.00 ± 0 vs. 1.09 ± 0.07 vs. 0.86 ± 0.06), (*p* < 0.05).

To sum up, the developmental totipotency genes of Oct4 and Rex01 and antiapoptotic gene of Bcl-2 had significant higher expressions at the penetration speed of 50 μm/s compared to those of the other groups, which implied oocyte penetration at the speed of 50 μm/s caused higher development potential. The higher totipotency and antiapoptotic gene expressions of the embryos verified the effectiveness of the optimized penetration speed at genetic level.

## 4. Discussion

In this paper, the developmental totipotency genes and an antiapoptotic gene were used to imply that the optimized penetration speed could cause higher development potential at genetic level. Additionally, the developmental potential (reflected by the cleavage rate) in this paper was consistent with some previous results: Liu et al. investigated the cell damage during microinjection by counting the number of ruptured bonds [19]. They found that the smaller penetration velocity generated more ruptured bonds. Thus, the larger penetration velocity caused lower cell damage, and the cells had better developmental potential, which was consistent with our results.

However, the mechanism of intracellular damage caused by micromanipulation remains unclear. Meanwhile, the damage during micromanipulation may be further reduced if a control algorithm is added. We will work on dynamic modeling of penetration process and explore a better penetration strategy in the future.

## 5. Conclusions

In this paper, we optimized the oocyte penetration speed based on the intracellular strain. We firstly analyzed the intracellular strain under different penetration speeds and performed the penetration experiments on porcine oocytes. Secondly, we studied the cell development potential after penetration at different penetration speeds. The statistical results showed that the percentage of large intracellular strain values decreased by 80% and the maximum and average intracellular strain values decreased by 25–38% at the penetration speed of 50 μm/s, compared to those at 10 μm/s, which reduced potential intracellular damage greatly. The results showed that the optimal penetration speed improved the cleavage rate from 65% to 86%. Finally, we verified the gene expression of oocytes after penetration at different speeds. The experimental results showed that the totipotency and antiapoptotic of oocytes were significantly higher after penetration at the speed of 50 μm/s, which verified the effectiveness of the optimization method at the gene level. Collectively, the results of the current investigation aimed to devise and optimize intracellular strain-related parameters that turned out to vary depending on different speeds of penetrating the porcine oocytes with the use of robotic (i.e., motorized) micro-manipulators might contribute to improvement of the efficiency of a variety of modern assisted reproductive technologies (ARTs). Taking the above-mentioned findings into consideration, the augmented efficiencies of microsurgical in vitro fertilization by ICSI and cloning by somatic cell nuclear transfer (SCNT) will be of especially great importance not only in pigs but also in other mammalian species [25,26,27,28,29,30,31].

## Figures and Tables

**Table 1:** Primers sequences and conditions for RT-qPCR.

Genes Primer	Sequences (5–3)	Size of PCR Products (bp)	Tm (°C)
Oct4	GCTCACTTTGGGGGTTCTCT TGAAACTGAGCTGCAAAGCC	80	59
Rex01	CTTCAAGGAGAGCGCAAAAC TGTCCCCAATCAAAAATGCT	299	52
Bcl-2	GCAACCCATCCTGGCACCT AACTCATCGCCCGCCTCCCT	133	60

## Data Availability

Not applicable.

## Supplementary Materials

The following supporting information can be downloaded at: https://www.mdpi.com/article/10.3390/mi13020309/s1, Videos S1–S6: Oocyte penetration processes at the penetration speeds of 10 μm/s, 20 μm/s, 30 μm/s, 40 μm/s, 50 μm/s and 100 μm/s and corresponding strain distributions.

## Appendix A. Statistical Analysis of the Penetration Process

**Table A1:** Descriptions of the abbreviations used in the Appendix A.

i ∈ 1, 2, 3, 4, 5, 6, 7, 8, 9, 10	Oocyte Number in One Group.
j ∈ 10, 20, 30, 40, 50, 100	Group that represents the oocytes were penetrated at the speeds of 10, 20, 30, 40, 50, and 100 μm/s.
n = 10	Number of the oocytes in one group.
N	Number of the points in each oocyte.
Strain_pene^i,j^	Strain distribution of oocyte i in group j in penetration process.
Max_p__strain_pene^i,j^	Max value of Strain_pene^i,j^ in each oocyte.
Avg_i__max_p__strain_pene^j^	Average value of Max_p__strain_pene^i,j^.
STD_i__max_p__strain_pene^j^	STD value of Max_p__strain_pene^i,j^.
Avg_p__strain_pene^i,j^	Average value of Strain_pene^i,j^ in each oocyte.
Avg_i__avg_p__strain_pene^j^	Average value of Avg_p__strain_pene^i,j^.
STD_i__avg_p__strain_pene^j^	STD value of Avg_p__strain_pene^i,j^.
Strain_pene^i,j,t^	Strain distribution of oocyte i in group j in frame t in penetration process.
Max_p__strain_pene^i,j,t^	Max value of Strain_pene^i,j^ in each oocyte in each frame.
Max_t__max_p__strain_pene^i,j^	Max value of Max_p__strain_pene^i,j,t^ in all frames in each oocyte.
Avg_i__max_t__max_p__strain_pene^j^	Average value of Max_t__max_p__strain_pene^i,j^ in each group.
Avg_p__strain_pene^i,j,t^	Average value of Strain_pene^i,j^ in each oocyte in each frame.
Avg_t__avg_p__strain_pene^i,j^	Average value of Avg_p__strain_pene^i,j,t^ in all frames in each oocyte.
Avg_i__avg_t__avg_p__strain_pene^j^	Average value of Avg_t__avg_p__strain_pene^i,j^ in each group.

We calculated the max strain in the last frame in the penetration process in three steps:

(1) Obtain the strain distribution in the last frame before the oocyte penetrated. The strain distribution of oocyte i in group j was defined as Strain_pene^i,j^.
(2) Obtain the max value of Strain_pene^i,j^ in each oocyte:
  $${\mathrm{Max}}_{p}{\_\mathrm{strain}\_\mathrm{pene}}^{i,j}=\underset{x,y}{\max}\left({\mathrm{Strain}\_\mathrm{pene}}^{i,j}\left(x,y\right)\right)$$
  where (x, y) represents the position in the oocyte.
(3) Obtain the average value and standard deviation (STD) of Max_p__strain_pene^i,j^ in each group:
  $${\mathrm{Avg}}_{i}{\_\max}_{p}{\_\mathrm{strain}\_\mathrm{pene}}^{j}=\frac{1}{n}\sum_{i=1}^{n}{\mathrm{Max}}_{p}\_\mathrm{strain}\_{\mathrm{pene}}^{i,j}$$
  $$\begin{array}{l} {\mathrm{STD}}_{i}{\_\max}_{p}{\_\mathrm{strain}\_\mathrm{pene}}^{j}=\\ {\;(\frac{1}{n-1}\sum_{i}^{}\left({\mathrm{Max}}_{p}{\_\mathrm{strain}\_\mathrm{pene}}^{i,j}-{\mathrm{Avg}}_{i}{\_\max}_{p}{\_\mathrm{strain}\_\mathrm{pene}}^{j}\right))}^{\frac{1}{2}} \end{array}$$
  where n represents the number of oocytes in one group.

We calculated the average strain in the last frame in two steps:

(1) Obtain the average value of Strain_pene^i,j^ in each oocyte:
  $${\mathrm{Avg}}_{p}{\_\mathrm{strain}\_\mathrm{pene}}^{i,j}=\frac{1}{N}\sum_{x,y}{\mathrm{Strain}\_\mathrm{pene}}^{i,j}\left(x,y\right)$$
  where N represents the number of the points in the oocyte.
(2) Obtain the average value and STD of Avg_p__strain_pene^i,j^ in each group:
  $${\mathrm{Avg}}_{i}{\_\mathrm{avg}}_{p}{\_\mathrm{strain}\_\mathrm{pene}}^{j}=\frac{1}{n}\sum_{i}{\mathrm{Avg}}_{p}{\_\mathrm{strain}\_\mathrm{pene}}^{i,j}$$
  $$\begin{array}{l} {\mathrm{STD}}_{i}{\_\mathrm{avg}}_{p}{\_\mathrm{strain}\_\mathrm{pene}}^{j}=\\ {\;(\frac{1}{n-1}\sum_{i}^{}\left({\mathrm{Avg}}_{p}{\_\mathrm{strain}\_\mathrm{pene}}^{i,j}-{\mathrm{Avg}}_{i}{\_\mathrm{avg}}_{p}{\_\mathrm{strain}\_\mathrm{pene}}^{j}\right))}^{\frac{1}{2}} \end{array}$$

We calculated the max strain in the penetration process in four steps:

(1) Obtain the strain distribution in each frame of the penetration process. The strain distribution of oocyte i in group j in frame t was defined as strain_pene^i,j,t^.
(2) Obtain the max value of strain_pene^i,j,t^ in each oocyte in each frame:
  $${\mathrm{Max}}_{p}{\_\mathrm{strain}\_\mathrm{pene}}^{i,j,t}=\underset{x,y}{\max}\left({\mathrm{strain}\_\mathrm{pene}}^{i,j,t}\left(x,y\right)\right)$$
  where (x, y) represents the position in the oocyte.
(3) Obtain the max value of Max_p__strain_pene^i,j,t^ in all frames in each oocyte:
  $${\mathrm{Max}}_{t}{\_\max}_{p}{\_\mathrm{strain}\_\mathrm{pene}}^{i,j}=\underset{t}{\max}\left({\mathrm{Max}}_{p}{\_\mathrm{strain}\_\mathrm{pene}}^{i,j,t}\right)$$
(4) Obtain the average value and STD of Max_p__strain_pene^i,j^ in each group:
  $${\mathrm{Avg}}_{i}{\_\max}_{t}{\_\max}_{p}{\_\mathrm{strain}\_\mathrm{pene}}^{j}=\frac{1}{n}\sum_{i}{\mathrm{Max}}_{t}{\_\max}_{p}{\_\mathrm{strain}\_\mathrm{pene}}^{i,j}$$
  $$\begin{array}{l} {\mathrm{STD}}_{i}{\_\max}_{t}{\_\max}_{p}{\_\mathrm{strain}\_\mathrm{pene}}^{j}=\\ {\;(\frac{1}{n-1}\sum_{i}\left({\mathrm{Max}}_{t}{\_\max}_{p}{\_\mathrm{strain}\_\mathrm{pene}}^{i,j}-{\mathrm{Avg}}_{i}{\_\max}_{t}{\_\max}_{p}{\_\mathrm{strain}\_\mathrm{pene}}^{j}\right))}^{\frac{1}{2}} \end{array}$$
  where n represents the number of oocytes in one group.

We calculated the average strain in the penetration process in three steps:

(1) Obtain the average value of strain_pene^i,j,t^ in each oocyte in each frame:
  $${\mathrm{Avg}}_{p}{\_\mathrm{strain}\_\mathrm{pene}}^{i,j,t}=\frac{1}{N}\sum_{x,y}{\mathrm{strain}\_\mathrm{pene}}^{i,j,t}\left(x,y\right)$$
(2) Obtain the average value of Avg_p__strain_pene^i,j,t^ in all frames in each oocyte:
  $${\mathrm{Avg}}_{t}{\_\mathrm{avg}}_{p}{\_\mathrm{strain}\_\mathrm{pene}}^{i,j}=\frac{1}{T}\sum_{t}{\mathrm{Avg}}_{p}{\_\mathrm{strain}\_\mathrm{pene}}^{i,j,t}$$
  where T represents the number of the frames in the penetration process.
(3) Obtain the average value and STD of Avg_p__strain_pene^i,j^ in each group:
  $${\mathrm{Avg}}_{i}{\_\mathrm{avg}}_{t}{\_\mathrm{avg}}_{p}{\_\mathrm{strain}\_\mathrm{pene}}^{j}=\frac{1}{n}\sum_{i}{\mathrm{Avg}}_{t}{\_\mathrm{avg}}_{p}{\_\mathrm{strain}\_\mathrm{pene}}^{i,j}$$
  $$\begin{array}{l} {\mathrm{STD}}_{i}{\_\mathrm{avg}}_{t}{\_\mathrm{avg}}_{p}{\_\mathrm{strain}\_\mathrm{pene}}^{j}=\\ {\;(\frac{1}{n-1}\sum_{i}\left({\mathrm{Avg}}_{t}{\_\mathrm{avg}}_{p}{\_\mathrm{strain}\_\mathrm{pene}}^{i,j}-{\mathrm{Avg}}_{i}{\_\mathrm{avg}}_{t}{\_\mathrm{avg}}_{p}{\_\mathrm{strain}\_\mathrm{pene}}^{j}\right))}^{\frac{1}{2}} \end{array}$$
